# Cordyceps Polysaccharides Attenuate Experimental Ischemic Stroke Injury: Association with IL-17/NF-κB Signaling

**DOI:** 10.3390/ijms27209035

**Published:** 2026-10-12

**Authors:** Huizhang Wang, Yifan Chen, Yang Jiang, Yan Liao, Saiwen Xi, Xia Li, Huiming Qi, Yihui Ma, Yutong Li, Mujie Fang, Changhua Shi, Cong Gai, Yibo Tang

**Affiliations:** 1 School of Traditional Chinese Medicine, Beijing University of Chinese Medicine, Beijing 102488, China; wanghuizhang26@163.com (H.W.); cyf20000807@163.com (Y.C.); yjiang2010@163.com (Y.J.); liaoyanbucm@163.com (Y.L.); lixiasd07@163.com (X.L.); 15515982884@163.com (H.Q.); yh2661888@163.com (Y.M.); 18801108584@163.com (Y.L.); mujie_fang@163.com (M.F.); shichanghua520@sina.com (C.S.); 2 School of Chinese Materia Medica, Beijing University of Chinese Medicine, Beijing 102488, China; 18811566952@163.com

**Keywords:** cordyceps polysaccharides, ischemic stroke, differential gene expression analysis, oxygen–glucose deprivation, IL-17A, NF-κB

## Abstract

Cordyceps polysaccharides (CSP) have shown protective potential in ischemic stroke, but their central inflammatory mechanisms remain unclear. This study examined whether the effects of CSP are associated with interleukin-17 (IL-17)/nuclear factor kappa B (NF-κB)-related signaling. Publicly available human transcriptomic datasets were integrated and analyzed to identify inflammation-related pathways in ischemic stroke, and a public mouse middle cerebral artery occlusion (MCAO) single-cell RNA sequencing (RNA-seq) dataset was analyzed to identify relevant brain cell populations. IL-17 receptor A (IL-17RA) knockdown was performed in oxygen–glucose deprivation (OGD)-exposed SH-SY5Y cells. The effects of CSP were further evaluated in MCAO rats and OGD-exposed BV-2 cells. Bioinformatics analyses showed enrichment of IL-17 and NF-κB signaling in ischemic stroke datasets, and single-cell module-score analysis showed that microglia exhibited the largest MCAO-associated increase in IL-17/NF-κB-related module scores. IL-17RA knockdown improved the viability of OGD-exposed SH-SY5Y cells. In MCAO rats, CSP improved neurological and behavioral outcomes, increased cerebral blood flow, and reduced cerebral edema, infarct volume, neuronal loss, microglial responses, and IL-17A/IL-17RA expression. In OGD-exposed BV-2 cells, CSP reduced IL-17A, IL-17RA, NF-κB activator 1 (ACT1), tumor necrosis factor receptor-associated factor 6 (TRAF6), IL-1β, IL-6, and the phosphorylated p65/total p65 ratio. These findings suggest that the protective effects of CSP in ischemic stroke models are associated with attenuation of IL-17/NF-κB-related neuroinflammation.

## 1. Introduction

Ischemic stroke (IS) is the most common form of stroke worldwide and remains a major contributor to global mortality and disability. In 2021, an estimated 7.8 million new cases of IS were reported globally, reflecting a substantial and growing public health burden over the past three decades [1]. At present, the primary therapeutic strategies for IS focus on restoring cerebral blood flow through reperfusion therapies, including intravenous thrombolysis with tissue plasminogen activator (tPA) and endovascular thrombectomy [2,3]. These interventions have significantly improved clinical outcomes in eligible patients. However, strict therapeutic time windows and the high cost of treatment continue to limit timely access to effective care for a substantial proportion of patients. Therefore, the development of novel neuroprotective strategies and pharmacological agents capable of increasing resistance to ischemic injury remains of significant clinical and public health importance.

Following the onset of IS, reduced cerebral blood flow caused by arterial stenosis or occlusion leads to insufficient oxygen and glucose supply, resulting in impaired energy metabolism and disruption of ionic homeostasis. These events ultimately trigger calcium overload and glutamate-mediated excitotoxicity, which contribute to neuronal necrosis and apoptosis [4]. Subsequently, complex neuroinflammatory responses rapidly develop within the ischemic core and surrounding regions. Activated microglia and astrocytes release a variety of proinflammatory mediators, including tumor necrosis factor-α (TNF-α), interleukin-1β (IL-1β), and interleukin-6 (IL-6), which promote infiltration of peripheral immune cells and exacerbate local inflammatory reactions [5,6]. Meanwhile, increased permeability of the blood–brain barrier (BBB) and leakage of inflammatory mediators further contribute to cerebral edema and aggravation of brain tissue injury [7]. Accumulating evidence indicates that excessive activation of inflammatory cascades is a key pathological mechanism responsible for secondary neuronal death in the ischemic penumbra and expansion of infarct volume [8,9,10].

Among the inflammatory pathways implicated in ischemic brain injury, interleukin-17 (IL-17) has attracted increasing attention as an important regulator of post-ischemic neuroinflammation. Previous studies have demonstrated that the levels of IL-17 are elevated in the brain tissue of rats following middle cerebral artery occlusion (MCAO), accompanied by an increase in cerebral infarct volume. Therefore, modulating the IL-17-mediated inflammatory signaling pathway may represent a potential therapeutic target for alleviating ischemic brain injury [11].

Polysaccharides derived from traditional Chinese medicinal plants (TCMPs) are recognized as important bioactive components in herbal medicine and have been widely reported to possess anti-inflammatory, antioxidant, and immunomodulatory properties [12,13]. Among these, Cordyceps polysaccharides (CSP), a major active constituent of Cordyceps sinensis, has demonstrated protective effects in a variety of experimental disease models, primarily through modulation of inflammatory and oxidative stress–related pathways [14,15]. For example, CSP has been shown to suppress the TLR4/NF-κB signaling pathway and regulate the Nrf2/HO-1 axis, thereby reducing inflammatory responses in different pathological conditions [16,17,18]. These findings suggest that CSP may exert neuroprotective effects in ischemic brain injury through regulation of inflammatory signaling pathways.

Our previous study demonstrated that CSP exerted preventive neuroprotective effects in experimental models of IS and provided preliminary insights into its mechanism of action [11]. Specifically, CSP was found to reduce IL-17 expression in MCAO rats by preserving intestinal barrier integrity and decreasing IL-23 expression. However, that study primarily focused on the role of the peripheral gut–inflammation axis in ischemic brain injury. Nevertheless, whether the protective effects of CSP against ischemic brain injury are associated with the regulation of IL-17/NF-κB signaling in the central nervous system remains unclear. Therefore, further investigation from the perspective of central neuroinflammatory mechanisms is warranted to better define the role of CSP in ischemic brain injury.

Collectively, our previous work showed that CSP protects against experimental ischemic stroke, whereas the central inflammatory signaling associated with this effect remains unclear. Given the involvement of IL-17 signaling and NF-κB activation in post-ischemic neuroinflammation, we focused on the IL-17/NF-κB-related inflammatory axis as a candidate mechanism. Therefore, the present study aimed to determine whether CSP attenuates ischemic brain injury in vivo and in vitro and whether this protection is accompanied by changes in IL-17/NF-κB-related inflammatory signaling. Transcriptomic analysis, single-cell analysis, interleukin-17 receptor A (IL-17RA) knockdown, MCAO rat experiments, and oxygen–glucose deprivation (OGD)-induced cellular models were used to address this question.

## 2. Results

### 2.1. CSP Mitigates Neurological Dysfunction in Rats with MCAO

To evaluate the protective effect of CSP in the rat MCAO model, we conducted in-depth pharmacodynamic studies. The experimental schedule for CSP administration and permanent middle cerebral artery occlusion (pMCAO) modeling is illustrated in Figure 1A, while changes in body weight across the experimental groups are presented in Figure 1B. All groups displayed steady weight gain during the feeding phase. The Sham group showed no significant weight change before and after modeling, while the MCAO and CSP groups exhibited significant weight loss one day post-modeling. As shown in Figure 1C–E, the MCAO group exhibited significantly higher brain water content, brain index, and modified Neurological Severity Score (mNSS) score than the Sham group. In contrast, the CSP group showed reduced brain water content, brain index, and modified Neurological Severity Score (mNSS) score compared with the MCAO group, indicating improved neurological function (*p* < 0.05). As shown in Figure 1F–I, the open-field test results revealed that, compared with the Sham group, rats in the MCAO group exhibited a significant reduction in travel distance (*p* < 0.05) and a downward trend in the number of entries into the central zone (*p* > 0.05). In contrast, rats in the CSP group showed significantly increased travel distance and a higher number of entries into the central zone (*p* < 0.01) compared with the MCAO group.

### 2.2. CSP Improved Cerebral Blood Flow and Attenuated Ischemic Brain Damage in MCAO Rats

As shown in Figure 2A–D, cerebral blood flow was significantly lower, and infarct volume was significantly greater in the MCAO group than in the Sham group. Compared with the MCAO group, the CSP group showed markedly enhanced cerebral blood flow and reduced infarct volume. As evidenced by NeuN and Iba1 immunohistochemistry data in Figure 2E–J, compared with the Sham group, the number of NeuN-positive cells and the proportion of positive area in the ischemic penumbra were significantly reduced in the MCAO group. Compared with the MCAO group, the number of NeuN-positive cells and the proportion of positive area in the ischemic penumbra increased in the CSP group. Compared with the Sham group, the density of Iba1-positive cells in the ischemic penumbra increased in the MCAO group, while the average number of process branches per Iba1-positive cell decreased. Compared with the MCAO group, the density of Iba1-positive cells in the ischemic penumbra decreased in the CSP group, and the mean number of process branches per Iba1-positive cell increased.

### 2.3. Cell Counting Kit-8 (CCK-8) Concentration Screening and Selection of CSP Working Concentration

To determine the working concentration of CSP in OGD-modeled SH-SY5Y and BV-2 cells, CCK-8 concentration-screening assays were performed (Figure 3A,B). Compared with the Control group, OGD modeling significantly reduced the viability of both SH-SY5Y and BV-2 cells. CSP improved cell viability within an optimal concentration range, with maximal recovery observed at 200 μg/mL in both cell lines; higher concentrations showed a reduced protective trend. Therefore, 200 μg/mL was selected as the working concentration for subsequent in vitro experiments.

**Figure 3:**
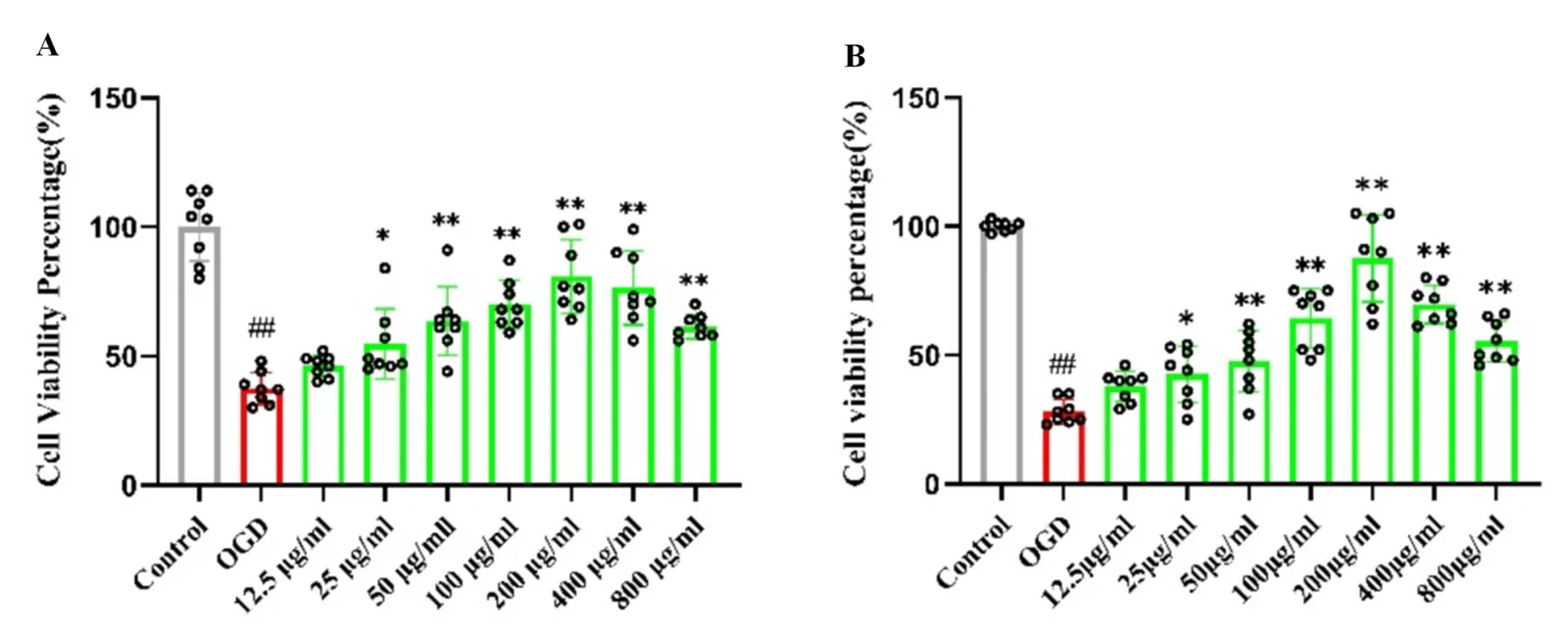
CCK-8-based concentration screening of CSP in OGD-treated SH-SY5Y and BV-2 cells. (**A**) Cell viability of SH-SY5Y cells treated with different concentrations of CSP under OGD conditions (*n* = 8). (**B**) Cell viability of BV-2 cells treated with different concentrations of CSP under OGD conditions (*n* = 8). Based on the concentration-response results, 200 μg/mL was selected for subsequent in vitro experiments. OGD, oxygen-glucose deprivation; CSP, Cordyceps polysaccharides. Results are expressed as mean ± SD; ^##^ *p* < 0.01 compared with the Control group; * *p* < 0.05 and ** *p* < 0.01 compared with the OGD group.

### 2.4. Kyoto Encyclopedia of Genes and Genomes (KEGG) and Gene Ontology (GO) Enrichment Analysis

To explore molecular pathways associated with IS and provide a basis for the subsequent pathway-focused experiments, we analyzed three publicly available transcriptomic datasets. Three IS-related datasets were retrieved from public databases, and principal component analysis (PCA) was conducted to assess sample distribution and batch effects. As shown in Figure 4A, the data points from different datasets were clustered according to batch, indicating substantial variation among samples and the presence of a significant batch effect. After batch effect correction, the expression profiles of the three datasets showed improved consistency and overlap, as illustrated in Figure 4B. Subsequently, genes shared in the intersection of the expression matrices from all three datasets were identified, and differential expression analysis was performed. As shown in Figure 4C,D, 57 differentially expressed genes (DEGs) were identified in the IS group compared with the Control group, including 41 significantly upregulated genes and 16 significantly downregulated genes. The volcano plot shows the overall distribution of the DEGs, whereas all 16 downregulated genes and the top 25 upregulated genes ranked by fold change were selected for heatmap visualization. Gene Ontology (GO) functional enrichment and Kyoto Encyclopedia of Genes and Genomes (KEGG) pathway enrichment analyses were subsequently performed on the identified DEGs. As presented in Figure 4E,F, both GO and KEGG enrichment analyses predominantly identified biological processes and pathways associated with immune responses and inflammatory reactions. Notably, KEGG pathway analysis revealed significant enrichment of inflammation-related signaling pathways, including the IL-17 and NF-κB signaling pathways, suggesting that these pathways may play important roles in the pathophysiology of IS.

### 2.5. Gene Set Enrichment Analysis (GSEA)

Notably, IL-17A has been reported to activate the NF-κB signaling pathway, thereby promoting the release of inflammatory cytokines. By integrating the pharmacological effects of CSP with the pathological mechanisms of IS, we hypothesized that the IL-17/NF-κB inflammatory axis may be involved in the protective effects of CSP in the IS model. To further examine this hypothesis, Gene Set Enrichment Analysis (GSEA) was performed for the IL-17 and NF-κB signaling pathways. As shown in Figure 5A,B, both IL-17 (normalized enrichment score (NES): 2.1, adjusted *p* < 0.001) and NF-κB (NES: 1.64, adjusted *p* = 0.02) signaling pathways were significantly enriched in the IS group, indicating a close association between these inflammatory signaling pathways and IS.

### 2.6. Knocking Down IL-17RA Increases the Cell Viability of OGD-Induced SH-SY5Y Cells

To evaluate the potential involvement of IL-17A/IL-17RA signaling in OGD-induced neuronal injury, we knocked down IL-17RA expression in SH-SY5Y cells and examined its effect on cell viability. The results shown in Figure 6A,B indicate that IL-17RA protein expression in the negative-control (NC) group showed no significant difference compared with the Control group. However, the siIL-17RA group exhibited significantly reduced IL-17RA protein expression, demonstrating effective IL-17RA knockdown. Figure 6C shows that there was no significant difference in cell viability between the siIL-17RA group and the Control group, whereas Figure 6D shows that cell viability in the OGD group was significantly reduced compared with the Control group. However, the OGD + siIL-17RA group displayed significantly higher cell viability than the OGD group.

### 2.7. CSP Attenuates MCAO-Associated Increases in IL-17A and IL-17RA Expression

To examine whether CSP-mediated protection was accompanied by changes in IL-17A/IL-17RA signaling, we investigated the expression of IL-17A and IL-17RA proteins in the ischemic penumbra of rats across all groups. As shown in Figure 7A–F, immunohistochemical results for IL-17A and IL-17RA revealed that, compared with the Sham group, the positive area and percentage of IL-17A- and IL-17RA-positive cells in the ischemic penumbra of MCAO rats were significantly increased. Compared with the MCAO group, the CSP group showed significantly smaller IL-17A- and IL-17RA-positive areas and lower percentages of positive cells in the ischemic penumbra. As shown in Figure 7G–J, Western blot analysis showed that IL-17A and IL-17RA protein expression was increased in the MCAO group compared with the Sham group and decreased in the CSP group compared with the MCAO group. The enzyme-linked immunosorbent assay (ELISA) results for IL-17A were consistent with the Western blot results.

### 2.8. Effects of CSP on IL-17/NF-κB-Axis Protein Expression in OGD-Treated BV-2 Cells

We evaluated IL-17A, IL-17RA, NF-κB activator 1 (ACT1), tumor necrosis factor receptor-associated factor 6 (TRAF6), IL-1β, and IL-6 expression, as well as the phosphorylated p65/total p65 (*p*-p65/p65) ratio, in OGD-treated BV-2 cells. As shown in Figure 8A–I, OGD significantly increased the expression of IL-17A, IL-17RA, ACT1, TRAF6, IL-1β, and IL-6 and elevated the *p*-p65/p65 ratio compared with the Control group. In contrast, CSP administration significantly reduced the expression of these proteins and the p-p65/p65 ratio compared with the OGD group. As shown in Figure 8J,K, IL-17RA fluorescence intensity was significantly higher in the OGD group than in the Control group, whereas CSP administration significantly reduced IL-17RA fluorescence intensity compared with the OGD group.

## 3. Discussion

The present study investigated the protective effects of Cordyceps polysaccharides (CSP) against experimental ischemic stroke (IS) and their relationship with IL-17/NF-κB signaling. CSP administration was initiated before permanent middle cerebral artery occlusion (pMCAO) and continued after modeling. CSP improved neurological and behavioral outcomes, reduced cerebral edema and infarction, preserved neuronal structure, and modulated the microglial response in the ischemic penumbra. Bioinformatics analyses identified IL-17 and NF-κB signaling as important inflammation-related pathways in IS. IL-17RA knockdown increased the viability of OGD-treated SH-SY5Y cells, while CSP reduced IL-17A and IL-17RA expression in ischemic brain tissue and attenuated IL-17/NF-κB-related inflammatory signaling in OGD-treated BV-2 cells. Collectively, these findings support the protective effects of CSP against ischemic injury and indicate that these effects are associated with attenuation of IL-17/NF-κB-related inflammatory signaling.

Cerebral ischemia results in a rapid decline in local blood supply, followed by energy failure, cerebral edema, neuronal dysfunction, and tissue infarction. In the present study, pMCAO caused significant neurological impairment, as reflected by increased mNSS scores, brain water content, and brain index. Rats in the MCAO group also exhibited reduced locomotor activity in the open-field test, consistent with impaired motor function after cerebral ischemia. CSP significantly improved these indices, indicating an overall reduction in acute ischemic brain injury. In particular, CSP increased the total distance traveled and the number of entries into the central area. The relatively high locomotor activity observed at 24 h may reflect a combined improvement in motor capacity and exploratory activity during the early stage after ischemic injury. Together with the improved mNSS scores, these behavioral findings support a beneficial effect of CSP on acute neurological performance.

The pathological findings further supported the protective effects of CSP. Under ischemic conditions, neurons in the ischemic core undergo rapid and largely irreversible injury, whereas neurons in the ischemic penumbra retain partial metabolic activity and remain responsive to protective intervention [19,20,21]. In addition, ischemia-related inflammatory responses and blood–brain barrier disruption contribute to neuronal loss and cerebral edema [22,23]. In this study, CSP increased cerebral blood flow in the affected hemisphere and reduced infarct volume. NeuN immunohistochemistry showed that CSP preserved NeuN-positive cells and neuronal structure in the ischemic penumbra. These changes were consistent with the improvement in neurological function and support the association between CSP administration and reduced acute ischemic brain injury.

Microglia are major cellular mediators of neuroinflammation after IS. Resting microglia generally exhibit small cell bodies and highly ramified processes, whereas ischemic injury induces changes in cell density, soma morphology, process length, and branching complexity [24]. Activated microglia can release inflammatory mediators such as IL-1β, TNF-α, and IL-6, which further influence neuronal survival and cerebral edema [25,26]. In the present study, pMCAO increased the density of Iba1-positive cells and reduced process branching in the ischemic penumbra. CSP administration decreased the density of Iba1-positive cells and improved microglial morphology, as reflected by more ramified processes and increased process branching. Previous studies have reported that naturally derived polysaccharides can regulate microglial inflammatory responses through pathways including NF-κB and MAPK signaling [27]. In line with these observations, our findings suggest that CSP administration is associated with attenuation of ischemia-associated microglial activation and with a less inflammatory microenvironment in the ischemic penumbra.

The concentration-screening experiments provided further evidence for the cellular protective effects of CSP. OGD significantly reduced the viability of both SH-SY5Y neuronal-like cells and BV-2 microglial cells. CSP improved cell viability in both cell lines within an optimal concentration range, with the strongest protective effect observed at 200 μg/mL. The reduced protection at higher concentrations indicates that the effect did not increase continuously with concentration. The response of both SH-SY5Y and BV-2 cells to 200 μg/mL CSP provided an experimental basis for selecting this concentration for subsequent CSP-related experiments in BV-2 cells.

Following confirmation of the protective effects of CSP, bioinformatics analyses were used to explore inflammatory mechanisms associated with IS. Integration of GSE16561, GSE22255, and GSE58294 identified differentially expressed genes related mainly to immune regulation, cytokine activity, and inflammatory responses. GO and KEGG analyses highlighted IL-17 and NF-κB signaling, while GSEA further demonstrated significant enrichment of both pathways in IS. IL-17A binds to a receptor complex containing IL-17RA and IL-17RC and subsequently recruits ACT1 and TRAF6, leading to NF-κB activation and the production of inflammatory mediators [28]. The consistent results obtained from different enrichment approaches indicate that the IL-17/NF-κB axis is closely associated with the inflammatory response in IS and provide a molecular basis for the subsequent experimental investigations.

To functionally evaluate the involvement of IL-17 receptor signaling in ischemia-like neuronal injury, IL-17RA was knocked down in SH-SY5Y cells. Western blotting confirmed effective suppression of IL-17RA protein expression, while IL-17RA knockdown had no significant effect on cell viability under normal culture conditions. Under OGD conditions, however, IL-17RA knockdown significantly increased SH-SY5Y cell viability. This pattern indicates that IL-17RA-dependent signaling contributes specifically to cellular injury under ischemic stress rather than to basal cell survival. The improvement in viability following IL-17RA knockdown also provides functional support for the IL-17 pathway identified by the bioinformatics analyses. These findings support the functional relevance of IL-17RA-associated signaling in ischemia-like neuronal injury.

The in vivo findings further connected IL-17 signaling with the protective effects of CSP. IL-17A and IL-17RA expression increased markedly in the ischemic penumbra after pMCAO, as demonstrated by immunohistochemistry. Western blotting confirmed the changes in IL-17A and IL-17RA protein expression, and ELISA showed a corresponding increase in IL-17A levels in brain homogenates. CSP consistently reduced these MCAO-associated changes across the three detection methods. The agreement among the immunohistochemical, Western blot, and ELISA results strengthens the evidence that CSP treatment is associated with reduced IL-17A and IL-17RA expression in ischemic brain tissue. Together with the reductions in infarct volume, neuronal injury, and microglial responses, these findings suggest that the protective effects of CSP are closely associated with attenuation of IL-17A/IL-17RA-related inflammation.

The rationale for using BV-2 cells was further supported by our analysis of the public mouse MCAO single-cell RNA sequencing dataset GSE174574. The single-cell analysis showed that neutrophils had relatively high IL-17/NF-κB-related module scores in both groups, whereas microglia exhibited the largest MCAO-associated increase. These findings supported the relevance of microglia-related inflammatory responses in the present study. These findings support the relevance of microglia-related inflammatory responses in the present mechanistic framework, although the mouse single-cell dataset was used to guide cell-type interpretation and does not replace the rat in vivo evidence. The BV-2 cell experiments provided further evidence at the cellular level. OGD increased the expression of IL-17A, IL-17RA, ACT1, TRAF6, IL-1β, and IL-6 and elevated the p-p65/p65 ratio, indicating enhanced IL-17/NF-κB-related inflammatory signaling. CSP reduced the expression of these proteins and decreased the p-p65/p65 ratio. Immunofluorescence analysis also showed reduced IL-17RA fluorescence intensity following CSP administration, consistent with the Western blot findings. The coordinated changes in multiple pathway-related proteins indicate that CSP attenuated IL-17/NF-κB-related inflammatory signaling rather than affecting only an isolated inflammatory marker.

Post-ischemic IL-17A production has been closely associated with γδ T-cell responses, which are regulated in part by IL-23 [29]. Our previous study showed that CSP reduced IL-23 and IL-17 expression while preserving intestinal barrier integrity in an IS model [11]. These findings suggest that CSP may influence post-ischemic inflammation partly through systemic immunomodulatory mechanisms, including peripheral immune and gut–inflammation-related pathways. Microglia can also participate in IL-17-related inflammatory amplification through cytokine interactions involving IL-1β and IL-23 [30,31].

Based on the present in vivo and cellular findings, CSP may be associated with IL-17-related inflammatory responses at both systemic and central levels. The reductions in IL-17A and IL-17RA expression in ischemic brain tissue may reflect modulation of central neuroinflammatory responses, whereas the relationship with peripheral IL-23/IL-17 signaling described in our previous work suggests a possible systemic contribution. In OGD-treated BV-2 cells, CSP was associated with reduced IL-17RA/ACT1/TRAF6/NF-κB-related inflammatory signaling, supporting a microglia-related cellular component. These findings provide a possible connection between previously reported systemic immunomodulatory effects of CSP and its potential modulation of central neuroinflammatory responses under ischemic conditions [11].

Some aspects of the present study warrant further investigation. First, although CSP attenuated ischemia-associated changes in Iba1-positive microglia, microglial phenotypic changes were not further examined using M1- and M2-associated markers. Additional studies incorporating these markers may provide further insight into how CSP influences pro-inflammatory and anti-inflammatory microglial responses. Second, the current in vivo study focused on the effects of CSP in the MCAO model and did not include a Sham + CSP group. Therefore, the findings mainly reflect the effects of CSP under ischemic conditions, while its potential influence under normal physiological conditions remains to be explored. Third, IL-17RA knockdown supported the involvement of IL-17RA-dependent signaling in OGD-induced injury but was evaluated independently of CSP administration. Future studies combining CSP with IL-17RA knockdown or overexpression, as well as pharmacological modulation of NF-κB activity, may help clarify the contribution of IL-17/NF-κB signaling to CSP-associated protection and the regulatory relationship between CSP and this signaling axis. Overall, the present findings indicate that CSP alleviates experimental ischemic brain injury and that its protective effects are associated with attenuation of IL-17/NF-κB-related neuroinflammation.

## 4. Materials and Methods

### 4.1. Preparation and Administration of CSP

CSP was purchased from Shanghai Yuanye Bio-Technology Co., Ltd. (Shanghai, China; product no. S27812). According to the manufacturer’s product information, the preparation was supplied as a powder with a polysaccharide content of ≥50%, determined by ultraviolet spectroscopy. The product was readily soluble in water and poorly soluble in organic solvents, and it was stored at 2–8 °C under nitrogen before use. We did not perform independent batch-specific molecular weight or monosaccharide composition analysis in the present study. For in vivo experiments, CSP (20 g) was dissolved in deionized water by ultrasonication and adjusted to a final volume of 200 mL (0.1 g/mL). The oral dose of 1000 mg/kg was selected based on our previous study of the IL-23/IL-17 axis, in which this regimen showed protective effects in MCAO rats [11]. For in vitro experiments, 50 mg of CSP was dissolved in the corresponding complete or glucose-free culture medium by ultrasonication to prepare a 5 mg/mL stock solution. The solution was sterilized through a 0.22 μm membrane filter and stored at 4 °C. Before use, the stock solution was diluted to the required concentrations.

### 4.2. Animal Experiment Grouping, Modeling, and Drug Administration

Healthy adult male specific-pathogen-free Sprague–Dawley rats weighing approximately 190 g were obtained from Beijing Sibeifu Biotechnology Co., Ltd. (Beijing, China). (Laboratory Animal Production License No. SCXK [Jing] 2024-0001). Rats were housed at 22 °C and 50% relative humidity, with five rats per cage, and were allowed to acclimatize for three days before the experiments. The experimental protocol was reviewed and approved by the Animal Experiment Ethics Committee of Beijing University of Chinese Medicine (protocol code BUCM20250805-001).

A total of 66 rats were used in three experimental cohorts. Following acclimatization, rats within each cohort were randomly allocated to the Sham, MCAO, and CSP groups using a random number table. The first cohort consisted of 30 rats, with 10 rats initially allocated to each group. Following pMCAO modeling, one rat in the MCAO group and one rat in the CSP group died. Therefore, the final numbers included in the subsequent analyses were 10, 9, and 9 in the Sham, MCAO, and CSP groups, respectively. A second cohort of 18 rats, with six rats per group, was used for immunohistochemical analysis. A third cohort of 18 rats, with six rats per group, was used for ELISA and Western blot analyses. ELISA was performed using samples from all six animals in each group, whereas Western blotting was performed using samples from three animals per group. Each individual rat was considered an independent experimental unit.

Animals were identified using coded animal numbers to conceal group allocation. The investigator performing the surgical procedures was blinded to treatment allocation between the MCAO and CSP groups but was necessarily aware of whether a Sham or pMCAO procedure was being performed. Neurological scoring was independently performed by two investigators who were blinded to all group allocations. Rats were weighed daily and received the corresponding administration by oral gavage at 09:00. Rats received CSP or saline once daily for six consecutive days before MCAO. MCAO modeling was performed 1 h after the sixth gavage. After modeling, animals were maintained for 24 h, during which one additional gavage administration was performed. Tissue samples were collected 24 h after MCAO. Animals were anesthetized with isoflurane (RWD, R510-22-10) using an inhalation anesthetic machine. Following complete anesthesia, pMCAO was induced using an intraluminal filament technique with filament insertion via the common carotid artery, consistent with the Koizumi method as summarized by Li et al. [32]. After modeling, the rats were placed on a warming pad until they had recovered. Neurological function scores were tested upon awakening, after which food and water were made available again.

### 4.3. Cell Experiment Grouping, Modeling, and Drug Administration

Mouse BV-2 microglial cells (Wuhan Shang’en Biotechnology Co., Ltd., Wuhan, China; catalog no. 202300515shu-03) and human SH-SY5Y neuroblastoma cells (Wuhan Shang’en Biotechnology Co., Ltd.; catalog no. 20240430-2) were used for the in vitro experiments. BV-2 cells were used to investigate microglia-associated inflammatory responses and the effects of CSP on IL-17/NF-κB-related signaling under oxygen–glucose deprivation (OGD) conditions. SH-SY5Y cells were used as a neuronal-like model to evaluate OGD-induced reductions in cell viability and the functional effect of IL-17RA knockdown. Both cell lines were used for the CSP concentration-response assay.

For the concentration-response assay, BV-2 and SH-SY5Y cells were assigned to the Control, OGD, and CSP treatment groups. When cell confluence reached approximately 70%, cells in the CSP treatment groups were pretreated with the corresponding concentration of CSP for 12 h, whereas cells in the Control and OGD groups were maintained in the corresponding complete culture medium. The medium was subsequently removed. Control cells were maintained in complete medium under normoxic conditions. Cells in the OGD group were cultured in glucose-free medium, whereas cells in the CSP treatment groups were cultured in glucose-free medium containing the corresponding concentration of CSP. Cells in the OGD and CSP treatment groups were incubated for 6 h at 37 °C in an atmosphere containing 5% CO2 and 95% N2 [33,34,35].

To evaluate the functional involvement of IL-17RA signaling suggested by the bioinformatics analysis, IL-17RA was knocked down in SH-SY5Y cells. Four siRNAs targeting human IL-17RA (IL-17RA-homo-1263, IL-17RA-homo-315, IL-17RA-homo-1052, and IL-17RA-homo-1623) were synthesized by GenePharma Co., Ltd. (Shanghai, China). Cells were transfected according to the manufacturer’s instructions, and IL-17RA-homo-1052, which showed the highest knockdown efficiency, was selected for subsequent experiments. Three sets of experiments were performed. First, cells were divided into the Control, NC, and siIL-17RA groups, and IL-17RA protein expression was measured by Western blotting to confirm knockdown efficiency. Second, cell viability was compared between the Control and siIL-17RA groups under normal culture conditions using the CCK-8 assay to determine whether IL-17RA knockdown affected basal cell viability. Third, cells were divided into the Control, OGD, and OGD + siIL-17RA groups, and cell viability was assessed using the CCK-8 assay to determine whether IL-17RA knockdown protected SH-SY5Y cells against OGD-induced injury.

### 4.4. Bioinformatics Analysis

Three human ischemic stroke (IS) microarray datasets were obtained from the Gene Expression Omnibus (GEO): GSE16561 (39 IS and 24 control whole-blood samples), GSE22255 (20 IS and 20 control peripheral blood mononuclear cell samples), and GSE58294 (69 cardioembolic stroke and 23 control whole-blood samples) [36,37,38]. All analyses were performed in R version 4.3.2. Expression matrices and platform annotations were obtained using “GEOquery”. Probe IDs were converted to gene symbols, unannotated probes were removed, and multiple probes corresponding to the same gene were averaged. Each dataset was log2-transformed when necessary and normalized using normalizeBetweenArrays in “limma”. Genes shared by the three datasets were retained and the matrices were merged. Batch effects were corrected using ComBat in “sva”, with dataset source as the batch variable and disease status retained as the biological variable. Correction efficiency was assessed by principal component analysis (PCA). Differentially expressed genes were identified using “limma” with adjusted *p* value < 0.05 and |log2FC| > 0.585. Gene Ontology (GO), Kyoto Encyclopedia of Genes and Genomes (KEGG), and Gene Set Enrichment Analysis (GSEA) were performed using “clusterProfiler”, “enrichplot”, and “GseaVis”. Dataset characteristics are provided in Supplementary Materials.

The mouse MCAO single-cell RNA-sequencing dataset GSE174574, containing three Sham and three MCAO brain samples collected 24 h after modeling, was analyzed using Seurat [39,40]. Cells with fewer than 200 or more than 6000 detected genes, or more than 20% mitochondrial transcripts, were excluded. After normalization, 3000 highly variable genes were identified, and the data were scaled with total unique molecular identifier (UMI) counts and mitochondrial transcript percentages regressed out. PCA and Harmony correction were followed by clustering, uniform manifold approximation and projection (UMAP) visualization, and marker-based cell-type annotation. An IL-17/NF-κB-related module was predefined with reference to the KEGG mouse IL-17 and NF-κB pathways and related downstream inflammatory mediators [41]. The module included Il17ra, Il17rc, Traf3ip2, Traf6, Rela, Nfkb1, Nfkbia, Mapk1, Mapk3, Mapk14, Jun, Fos, Il6, Cxcl1, Cxcl2, Cxcl3, Cxcl5, Ccl2, Ccl3, Ccl4, Ccl7, Ccl12, Ptgs2, Mmp3, Mmp9, S100a8, S100a9, Lcn2, Csf3, and Tnf. Detectable genes were scored using Seurat AddModuleScore [42]. Sample-level mean scores were compared between the Sham and MCAO groups for each cell type, and Δ scores were calculated as the mean MCAO score minus the mean Sham score.

### 4.5. Laser Speckle Blood Flow Imaging

Twenty-four hours after modeling, the rats were anesthetized. A midline incision was made along the posterior neck region, extending down to the area below both eyes. The scalp was then retracted laterally to fully expose the skull. The Moor FLPI-2 laser speckle blood flow imaging system was activated to capture blood flow images and save the files. Moor FLPI-2 V4.0 software was launched to select the data files for analysis. Upon entering the data analysis interface, bilateral cerebral blood flow values were obtained.

### 4.6. 2,3,5-Triphenyltetrazolium Chloride Staining (TTC)

Following laser speckle blood flow imaging, the brains were rapidly removed. After carefully removing the cerebellum and brainstem, the whole brains were placed at −20 °C for approximately 20 min to facilitate sectioning. The brains were then cut into consecutive coronal slices with a thickness of approximately 2 mm. The slices were incubated in freshly prepared 1% 2,3,5-triphenyltetrazolium chloride (TTC) solution at 37 °C for 30 min in the dark, with gentle agitation to ensure uniform staining of the tissue. After staining, the slices were rinsed with cold phosphate-buffered saline (PBS) and fixed in 4% paraformaldehyde for subsequent analysis. Digital images of each brain slice were captured, and the infarcted areas were measured using ImageJ software (version 1.54u8; National Institutes of Health, Bethesda, MD, USA). The infarct volume for each slice was calculated by multiplying the infarct area by the slice thickness (2 mm), and the total infarct volume was obtained by summing the values from all slices. To minimize the influence of cerebral edema, the corrected infarct volume was calculated using the indirect method according to the following formula [43]:

[(contralateral hemisphere volume-undamaged ipsilateral hemisphere volume)/(contralateral hemisphere volume × 2)] × 100%.

### 4.7. Measurement of Brain Water Content

Brain water content was measured using the wet–dry weight method as previously described [44]. Fresh brain tissue was immediately weighed to obtain the wet weight and then dried in a constant-temperature oven at 60 °C until a constant weight was reached. The dried tissue was subsequently weighed to obtain the dry weight. Brain water content was calculated using the following formula:

Brain water content (%) = [(wet weight − dry weight)/wet weight] × 100%.

### 4.8. Measurement of Brain Index

Rats were euthanized 24 h after MCAO and the brain was rapidly removed. After removing the cerebellum and brainstem, surface moisture was gently blotted with filter paper, and the entire brain was immediately weighed to obtain the brain weight. The body weight of each rat was recorded at the same time. The brain index was calculated using the following formula [45]:

Brain index (%) = brain weight (g)/body weight (g) × 100%.

### 4.9. Modified Neurological Severity Score (mNSS)

Neurological deficits were evaluated using the modified Neurological Severity Score (mNSS). Assessments were performed after the rats had recovered from anesthesia and at 24 h after MCAO modeling. The scoring criteria are presented in Table 1, with higher scores indicating more severe neurological impairment [46].

### 4.10. Open-Field Test

At 24 h after MCAO modeling, each rat was individually placed in the center of the open-field apparatus and allowed to explore freely for 5 min in a quiet environment. Locomotor activity was recorded using a video camera and subsequently analyzed using SuperMaze software (version 3.3.0; Shanghai Xinruan Information Technology Co., Ltd., Shanghai, China). Behavioral parameters, including the total distance traveled, were recorded. After each trial, the apparatus was cleaned with 75% ethanol to minimize potential olfactory interference [47,48].

### 4.11. Paraffin Section Preparation

At the end of the experiment, rats were anesthetized and euthanized, and the brains were rapidly collected and rinsed with pre-chilled PBS. Brain tissues were fixed in 4% paraformaldehyde at 4 °C for 24 h, dehydrated through a graded ethanol series, cleared in xylene, and embedded in paraffin. The paraffin-embedded tissues were sectioned into 4 μm-thick slices using a rotary microtome (Leica RM2125 RTS; Leica Microsystems, Wetzlar, Germany). The sections were mounted on glass slides and dried at 60 °C before immunohistochemical analysis.

### 4.12. Immunohistochemistry (IHC)

Paraffin-embedded brain sections were deparaffinized in xylene and rehydrated through graded ethanol. Microwave-induced antigen retrieval was performed using ethylenediaminetetraacetic acid (EDTA) antigen retrieval buffer. After cooling to room temperature, the sections were blocked for 30 min using the blocking solution supplied with the immunohistochemistry kit (Proteintech, PK10006). The sections were then incubated overnight at 4 °C with primary antibodies against IL-17A (Proteintech, Rosemont, IL, USA; 26163-1-AP; rabbit; 1:200), IL-17RA (Abcam, Cambridge, UK; ab180904; rabbit; 1:200), Iba1 (Abcam; ab178846; rabbit; 1:200), or NeuN (Abcam; ab177487; rabbit; 1:200). Subsequently, the sections were incubated with a horseradish peroxidase (HRP)-conjugated anti-rabbit secondary antibody (ZSGB-BIO, PV-6001; Beijing, China) for 30 min at room temperature. Immunoreactivity was visualized using 3,3′-diaminobenzidine (DAB), followed by hematoxylin counterstaining, dehydration, clearing, and mounting. Corresponding ischemic penumbral regions were selected across groups using consistent histological criteria, and images were acquired under identical magnification and imaging settings. Positive cell numbers and positive area percentages were quantified using ImageJ software, and the number of process branches per Iba1-positive cell was manually counted. Statistical analyses were performed using GraphPad Prism 10.1.2. Each analyzed section was obtained from a different rat, and each rat was treated as an independent experimental unit.

### 4.13. Immunofluorescence (IF)

BV-2 cells were divided into the Control, OGD, and OGD + CSP groups, with six independent confocal culture dishes per group. Control cells were maintained under normal culture conditions, whereas cells in the OGD and OGD + CSP groups were exposed to glucose-free medium under hypoxic conditions for 6 h. CSP was administered as described above. Immediately after OGD, cells were washed with pre-chilled PBS, fixed with 4% paraformaldehyde for 30 min, permeabilized with 0.3% Triton X-100 for 10 min, and blocked with 5% bovine serum albumin for 30 min. Cells were incubated overnight at 4 °C with an anti-IL-17RA primary antibody (AFFINITY, DF3602; 1:300), followed by incubation with a fluorescently labeled secondary antibody (AFFINITY, S0006; 1:300) for 30 min at room temperature in the dark. After washing, the cells were mounted using 4′,6-diamidino-2-phenylindole (DAPI)-containing antifade mounting medium and imaged using a confocal laser scanning microscope.

### 4.14. Western Blotting

Protein samples from the ischemic cerebral hemisphere of MCAO rats, BV-2 cells, and SH-SY5Y cells were subjected to Western blot analysis. Equal amounts of total protein (20 μg per lane) were separated using 10% or 15% sodium dodecyl sulfate–polyacrylamide gel electrophoresis (SDS-PAGE) gels and transferred onto 0.22 μm polyvinylidene fluoride (PVDF) membranes. After blocking with 5% skimmed milk in Tris-buffered saline containing Tween 20 (TBST) for 2 h, the membranes were incubated overnight at 4 °C with primary antibodies against IL-17A (1:1000; Proteintech, Rosemont, IL, USA; 26163-1-AP), IL-17RA (1:1000; Proteintech; 32055-1-AP), TRAF6 (1:1000; Cell Signaling Technology, Danvers, MA, USA; 8025S), TRAF3IP2/ACT1 (1:1000; Proteintech; 26692-1-AP), p65 (1:1000; Proteintech; 10745-1-AP), p-p65 (1:1000; Cell Signaling Technology; 3033T), IL-1β (1:2000; ZhongHe, Beijing, China; ZHS5008), IL-6 (1:1000; Immunoway; YM8783), or β-actin (1:5000; Lableader, Beijing, China; A1101). β-actin was used as the loading control. After washing, the membranes were incubated with the appropriate HRP-conjugated anti-rabbit or anti-mouse secondary antibody (1:5000; NCM, Shanghai, China; P8001; P8002) for 1 h at room temperature. Protein bands were visualized using an enhanced chemiluminescence (ECL) substrate, and the chemiluminescent signals were captured for analysis.

### 4.15. Enzyme-Linked Immunosorbent Assay (ELISA)

Brain IL-17A levels were measured using an enzyme-linked immunosorbent assay (ELISA). Rats were divided into the Sham, MCAO, and CSP groups, with six animals per group. Brain tissues from the ischemic hemisphere were homogenized in radioimmunoprecipitation assay (RIPA) lysis buffer on ice and centrifuged at 12,000× *g* for 10 min at 4 °C. The supernatants were collected, and total protein concentrations were determined using a bicinchoninic acid (BCA) protein assay kit (Lableader, B5001). After normalization to the same protein concentration, IL-17A levels were measured using a commercial ELISA kit (Cloud-Clone Corp., Katy, TX, USA, SEA063Ra, 96T) according to the manufacturer’s instructions. Absorbance was measured at 450 nm using a microplate reader, and IL-17A levels were normalized to the total protein content of each sample.

### 4.16. Cell Counting Kit-8 (CCK-8)

Cell viability was assessed using a Cell Counting Kit-8 (CCK-8; Lableader, catalog no. CK001) according to the manufacturer’s instructions. BV-2 and SH-SY5Y cells were seeded in 96-well plates and subjected to the indicated treatments. Cell-free wells containing the corresponding culture medium were used as blanks, while unused peripheral wells were filled with sterile PBS to minimize evaporation. After treatment, 10 μL of CCK-8 reagent was added directly to each well containing 100 μL of culture medium, corresponding to a reagent-to-medium volume ratio of 1:10. The plates were incubated at 37 °C for 1 h, and optical density (OD) at 450 nm was measured using a microplate reader [49]. Cell viability was calculated using the following formula:

Cell viability (%) = [(OD treatment − OD blank)/(OD control − OD blank)] × 100%

### 4.17. Statistics

All data are presented as the mean ± standard deviation (SD), and the sample size for each experiment is indicated in the corresponding figure legends. Normality was assessed using the Shapiro–Wilk test. Normally distributed data involving multiple groups were analyzed using one-way analysis of variance (ANOVA). When treatment groups were compared with a designated control group, Dunnett’s multiple-comparison test was used for post hoc analysis. Data that did not meet the normality assumption were analyzed using the Kruskal–Wallis test followed by Dunn’s multiple-comparison test. For the in vivo experiments, the main comparisons were between the MCAO and Sham groups and between the CSP and MCAO groups. For the in vitro experiments, the main comparisons were between the OGD and Control groups and between the treatment and OGD groups. All tests were two-sided, and *p* < 0.05 was considered statistically significant. Statistical analyses were performed using GraphPad Prism 10.1.2.

## 5. Conclusions

This study indicates that CSP alleviates experimental ischemic brain injury, as evidenced by improvements in neurological function, cerebral blood flow, infarct volume, cerebral edema, neuronal preservation, and microglial responses in pMCAO rats. Bioinformatics analyses, single-cell evidence, and IL-17RA knockdown experiments support the involvement of IL-17/NF-κB-related inflammatory signaling in ischemia-like injury. Furthermore, CSP administration was associated with reduced IL-17A and IL-17RA expression in ischemic brain tissue and attenuation of IL-17/NF-κB-related inflammatory signaling in OGD-treated BV-2 cells. Collectively, these findings suggest that the protective effects of CSP against experimental IS are associated with attenuation of IL-17/NF-κB-related neuroinflammation and provide an experimental basis for further investigation of its molecular targets.

## Figures and Tables

**Figure 1:**
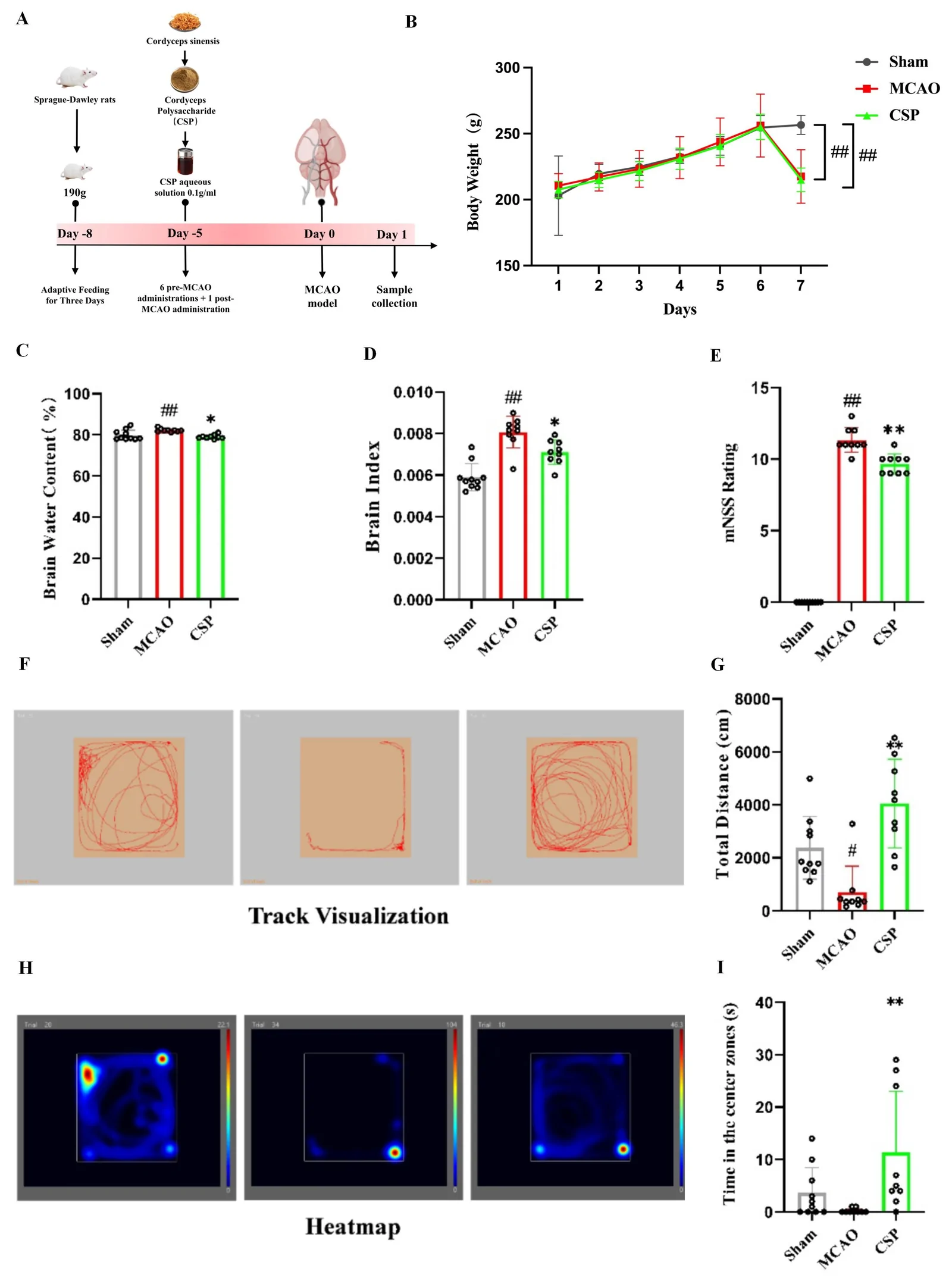
CSP alleviates cerebral injury in MCAO rats. (**A**) Experimental schedule showing CSP/saline administration for 6 days before MCAO and one additional administration during the 24 h post-MCAO period. (**B**) Changes in body weight of rats in each group before and after MCAO modeling. (**C**) Brain water content in each group. (**D**) Brain index in each group. (**E**) mNSS scores 1 day after modeling. (**F**) Representative locomotor tracks in the open-field test (OFT). (**G**) Total movement distance in the OFT. (**H**) Representative locomotor heatmaps in the OFT. (**I**) Number of entries into the central area in the OFT. (Sham *n* = 10, MCAO *n* = 9, CSP *n* = 9). MCAO, middle cerebral artery occlusion; CSP, Cordyceps polysaccharides; OFT, open-field test. Results are expressed as mean ± standard deviation (SD); ^#^ *p* < 0.05 and ^##^ *p* < 0.01 compared with the Sham group; * *p* < 0.05 and ** *p* < 0.01 compared with the MCAO group.

**Figure 2:**
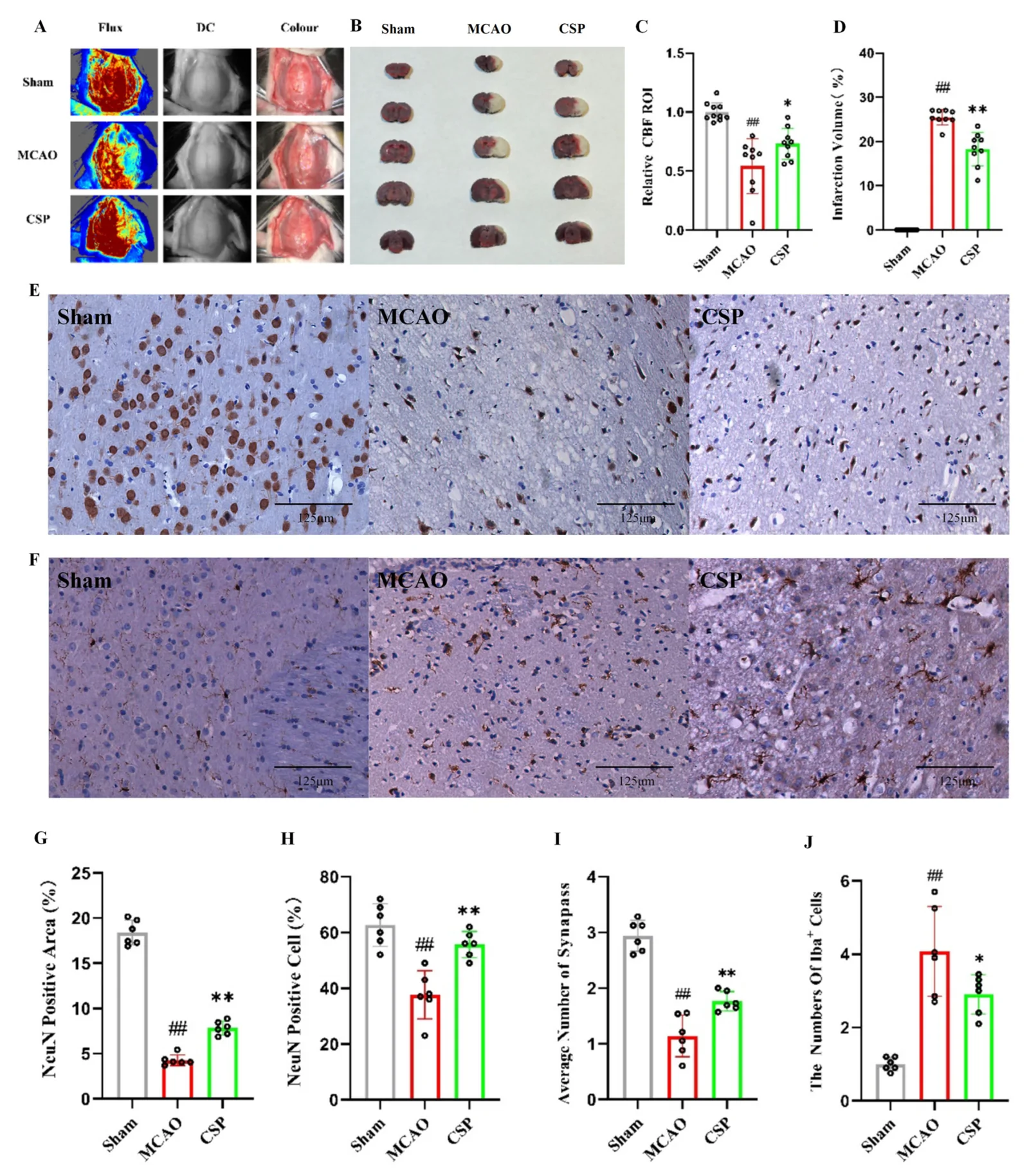
CSP improves morphological alterations in the brain tissue of MCAO rats. (**A**) Representative laser speckle blood-flow images. (**B**) Representative 2,3,5-triphenyltetrazolium chloride (TTC)-stained brain sections. (**C**) Ipsilateral cerebral blood flow measurements. (**D**) Cerebral infarct volume. ((**C**,**D**): Sham *n* = 10, MCAO *n* = 9, CSP *n* = 9). (**E**) NeuN immunohistochemistry in the ischemic penumbra. (**F**) Iba1 immunohistochemistry in the ischemic penumbra. (**G**) Percentage of NeuN-positive area. (**H**) Percentage of NeuN-positive cells. (**I**) Iba1-positive cell density in the ischemic penumbra. (**J**) Mean number of process branches per Iba1-positive cell in the ischemic penumbra. ((**G**–**J**): *n* = 6). MCAO, middle cerebral artery occlusion; CSP, Cordyceps polysaccharides. Results are expressed as mean ± SD; ^##^ *p* < 0.01 compared with the Sham group; * *p* < 0.05 and ** *p* < 0.01 compared with the MCAO group.

**Figure 4:**
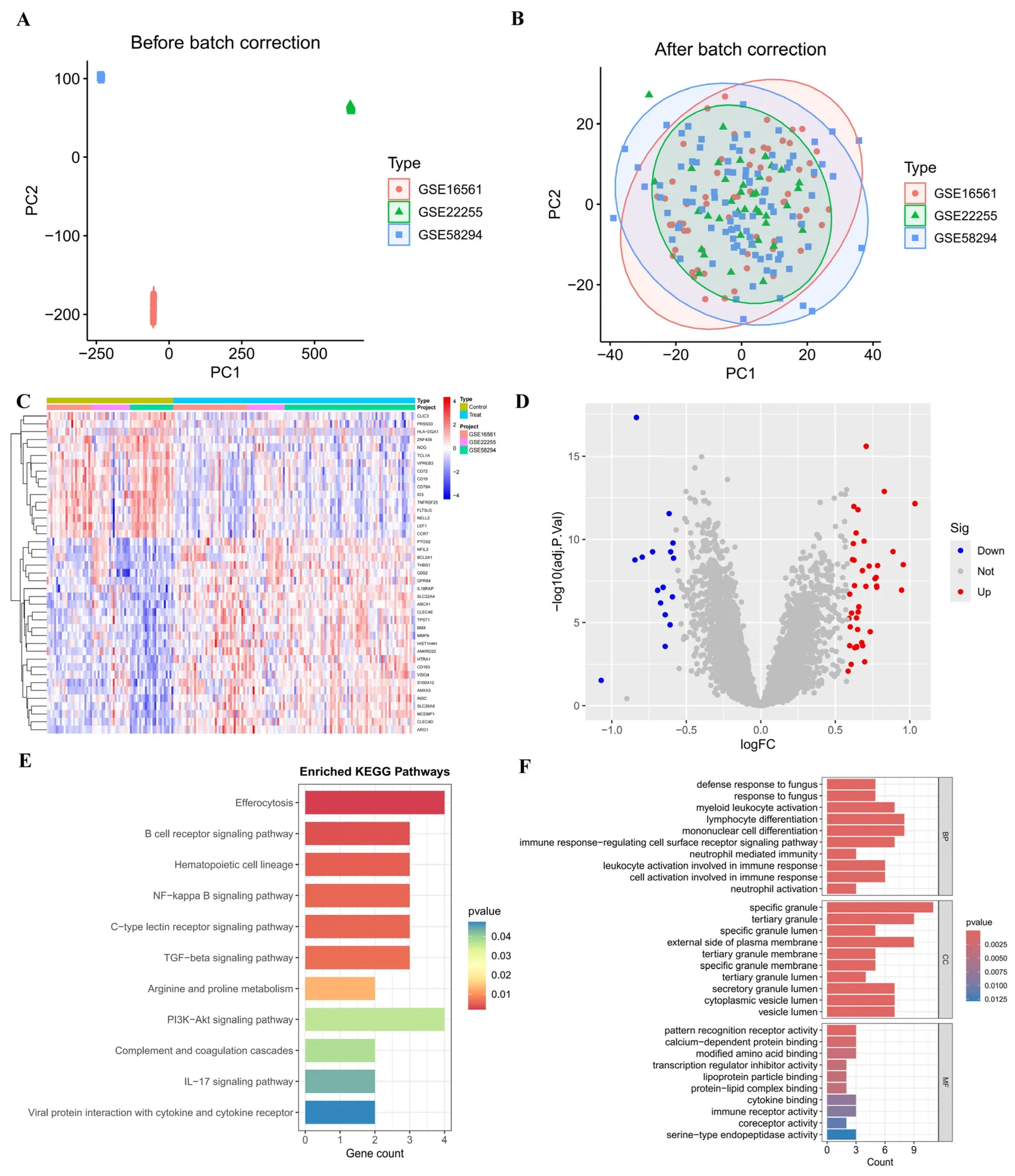
KEGG and GO enrichment analysis. (**A**) Principal component analysis before batch adjustment. (**B**) Principal component analysis after batch correction. (**C**) Heatmap of differentially expressed genes. (**D**) Volcano plot of differentially expressed genes. (adj. *p* value < 0.05 and |log2FC| > 0.585). (**E**) KEGG enrichment analysis bar chart. (**F**) GO enrichment analysis bar chart.

**Figure 5:**
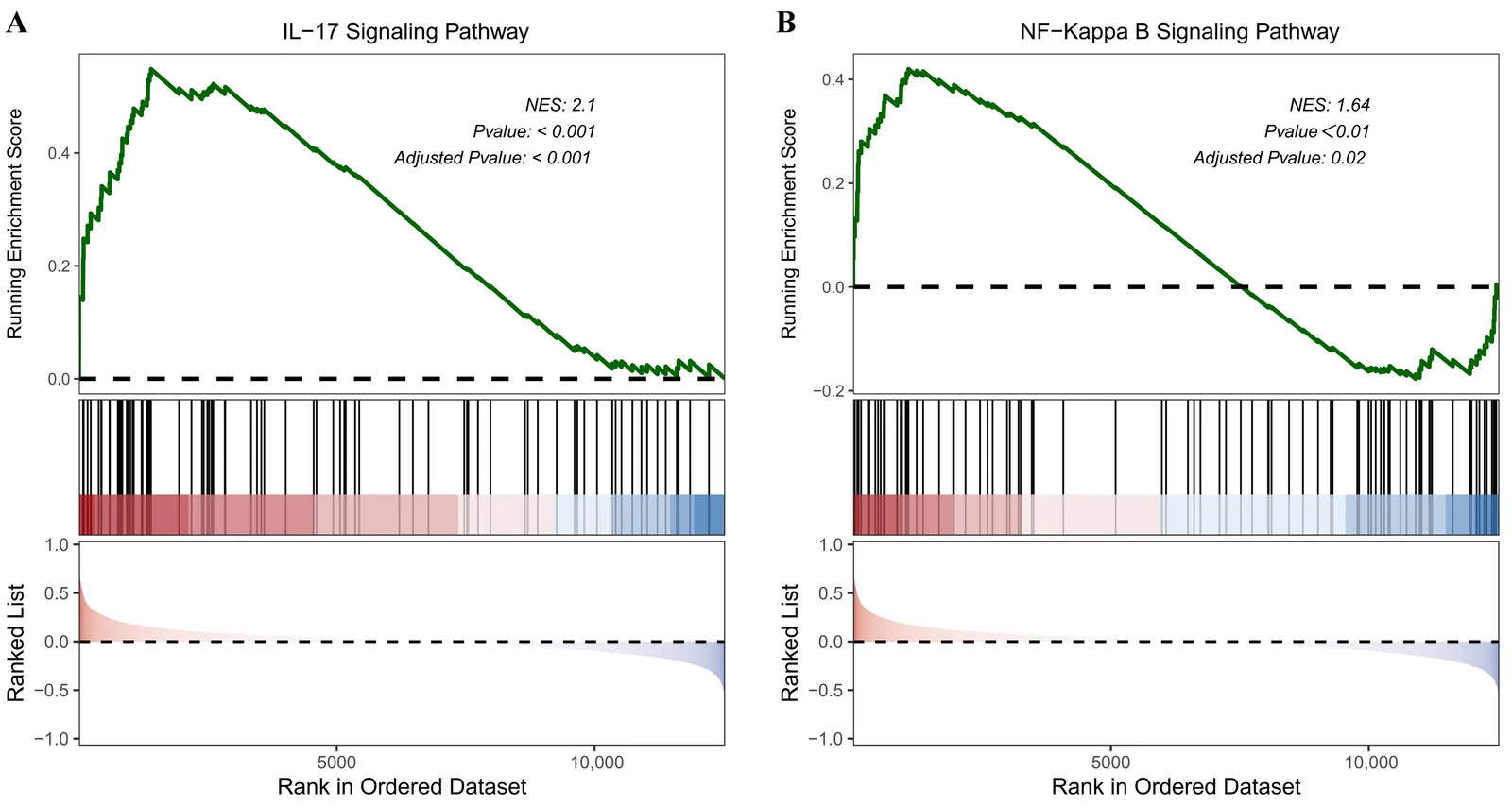
GSEA enrichment analysis. (**A**) IL-17 signaling pathway GSEA enrichment analysis diagram. (**B**) NF-κB signaling pathway GSEA enrichment analysis diagram.

**Figure 6:**
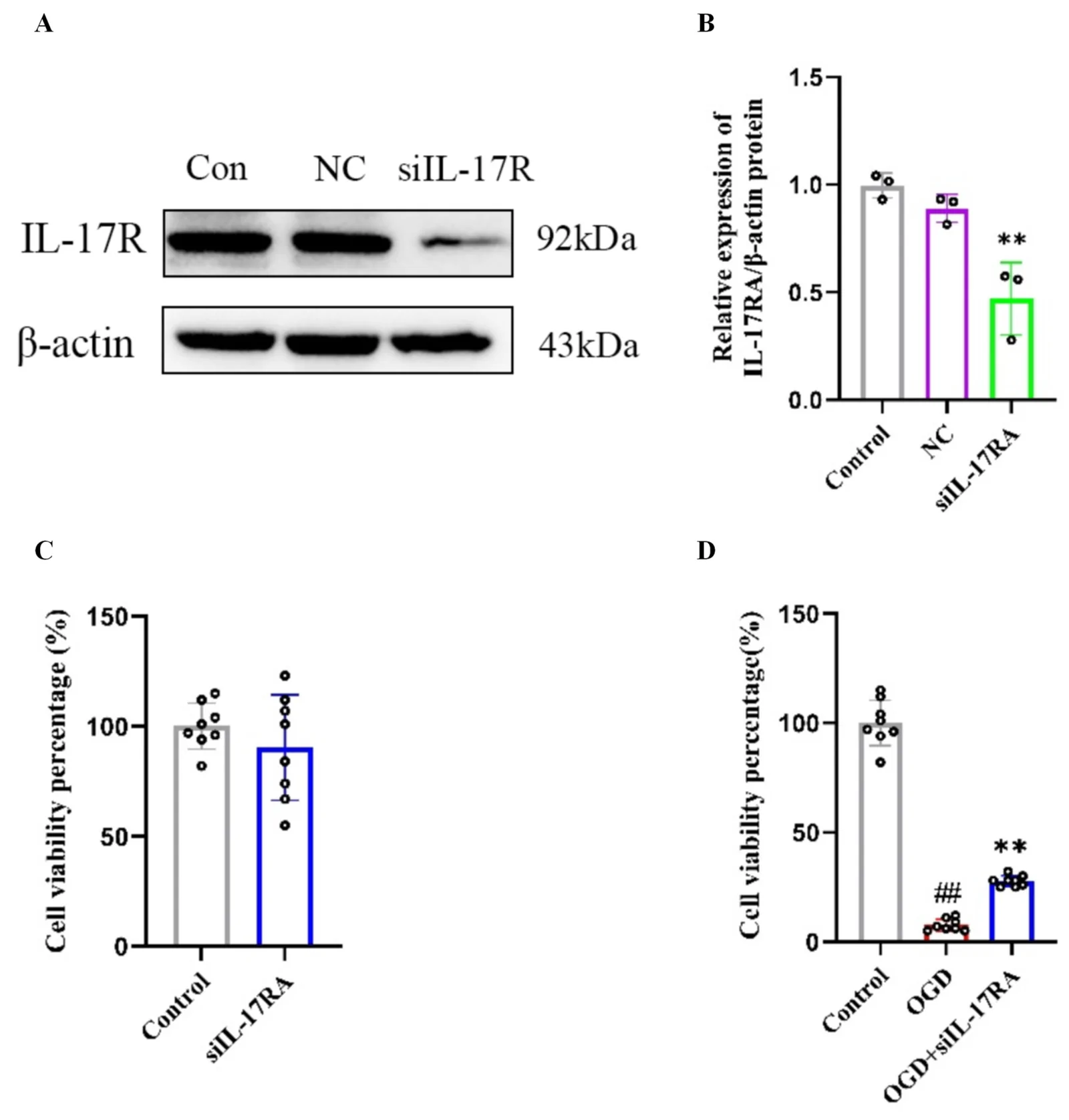
Knockdown of IL-17RA increases cell viability in OGD-modeled SH-SY5Y cells. (**A**) Representative Western blot images of IL-17RA and β-actin in the Control, negative control (NC), and siIL-17RA groups. (**B**) Quantification of IL-17RA protein expression normalized to β-actin (*n* = 3). (**C**) Cell viability in the Control and siIL-17RA groups (*n* = 8). (**D**) Cell viability in the Control, OGD, and OGD + siIL-17RA groups (*n* = 8). OGD, oxygen-glucose deprivation; NC, negative control; siIL-17RA, small interfering RNA targeting IL-17RA. Results are expressed as mean ± SD; ^##^ *p* < 0.01 compared with the Control group; ** *p* < 0.01 compared with the OGD or NC group, as indicated.

**Figure 7:**
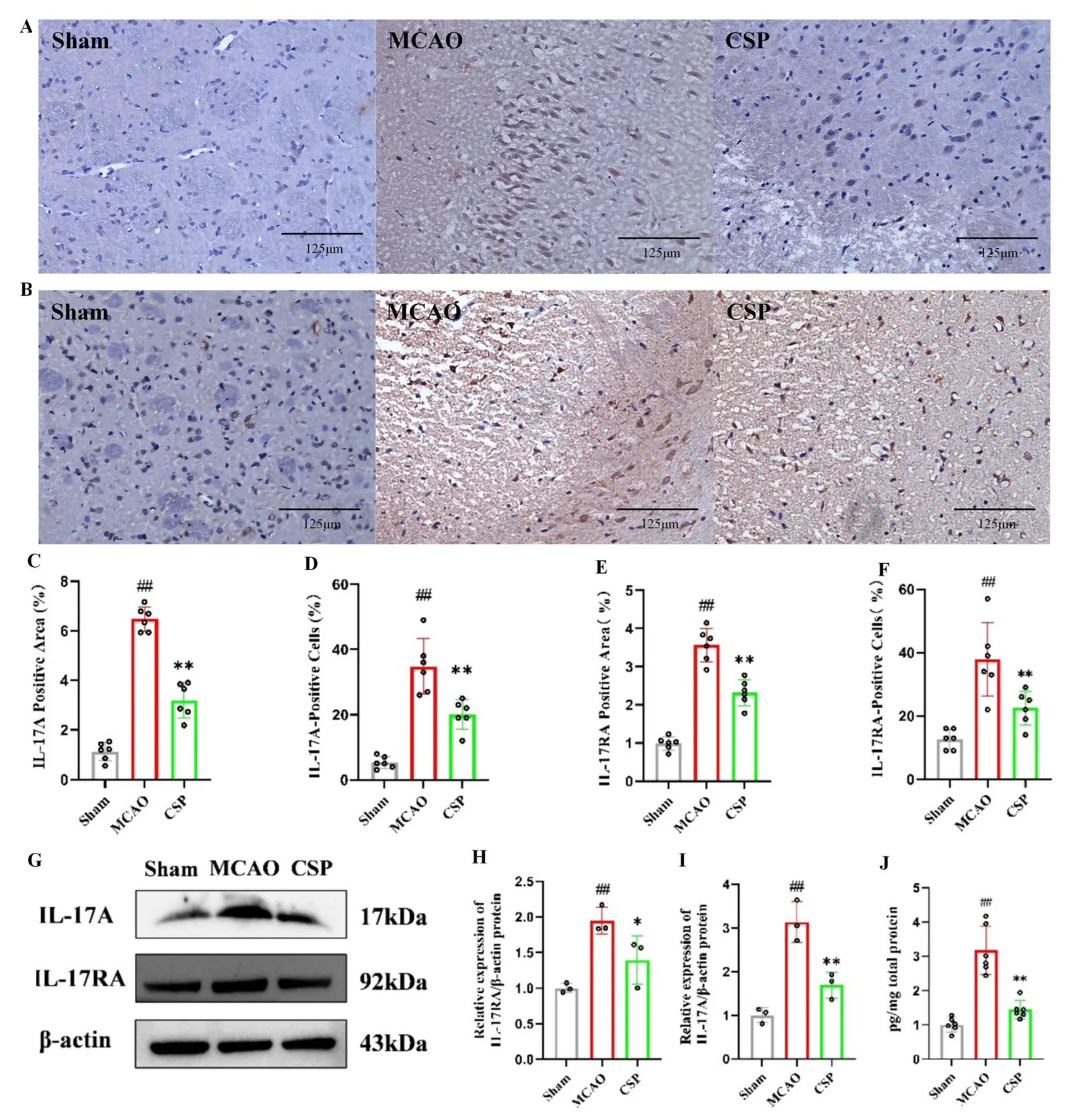
CSP attenuates MCAO-associated increases in IL-17A and IL-17RA expression in the ischemic penumbra. (**A**) IL-17A immunohistochemistry in brain tissue across groups. (**B**) IL-17RA immunohistochemistry in brain tissue across groups. (**C**) Percentage of IL-17A-positive area. (**D**) Percentage of IL-17A-positive cells. (**E**) Percentage of IL-17RA-positive area. (**F**) Percentage of IL-17RA-positive cells (*n* = 6). (**G**) Representative Western blot images of IL-17A, IL-17RA, and β-actin. (**H**) Quantification of IL-17A protein expression. (**I**) Quantification of IL-17RA protein expression (*n* = 3). (**J**) IL-17A levels in brain homogenates measured by ELISA (*n* = 6). MCAO, middle cerebral artery occlusion; CSP, Cordyceps polysaccharides. Results are expressed as mean ± SD; ^##^ *p* < 0.01 compared with the Sham group; * *p* < 0.05 and ** *p* < 0.01 compared with the MCAO group.

**Figure 8:**
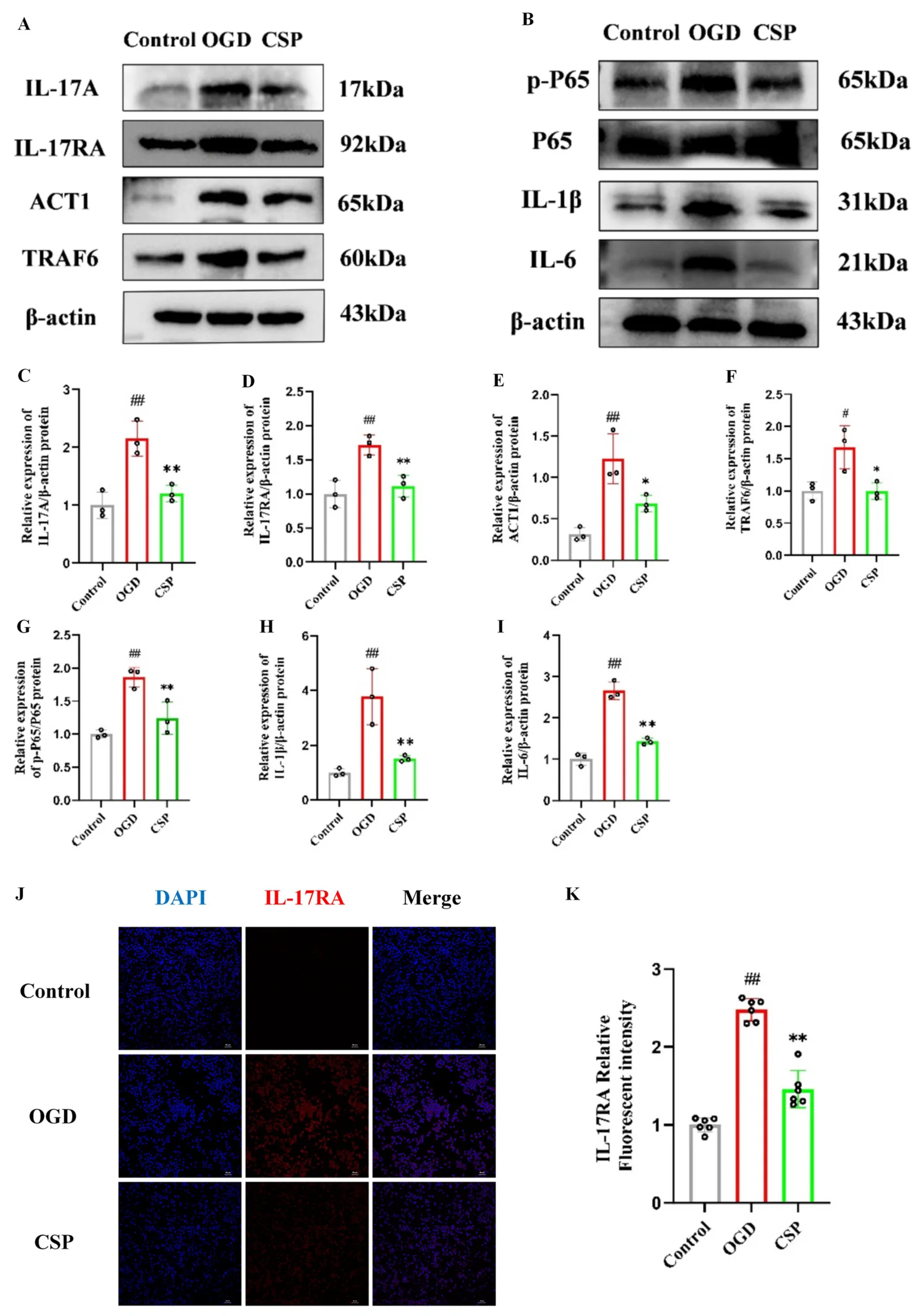
CSP attenuates IL-17/NF-κB-related inflammatory signaling in OGD-treated BV-2 cells. (**A**) Representative Western blot images of IL-17A, IL-17RA, ACT1, TRAF6, and β-actin in BV-2 cells. (**B**) Representative Western blot images of p-p65, p65, IL-1β, IL-6, and β-actin in BV-2 cells. (**C**–**I**) Quantification of IL-17A, IL-17RA, ACT1, TRAF6, the p-p65/p65 ratio, IL-1β, and IL-6 in BV-2 cells (*n* = 3). (**J**) Immunofluorescence labeling of IL-17RA in BV-2 cells. (**K**) Quantification of IL-17RA fluorescence intensity (*n* = 6). OGD, oxygen-glucose deprivation; CSP, Cordyceps polysaccharides. Results are expressed as mean ± SD; ^#^ *p* < 0.05 and ^##^ *p* < 0.01 compared with the Control group; * *p* < 0.05 and ** *p* < 0.01 compared with the OGD group.

**Table 1:** Modified neurological severity scores (mNSS).

Items	Points
**Motor tests**	**6**
Hanging rat by tail (0–3)	3
Forelimb flexion	1
Hindlimb flexion	1
Head deviation > 10° from the vertical axis within 30 s	1
**Placing rat on the floor (0–3)**	3
Normal walk	0
Inability to walk straight	1
Circling to the paretic side	2
Fall down to the paretic side	3
**Sensory tests**	**2**
Visual and tactile placing	1
Proprioceptive test (deep sensory)	1
**Beam balance tests**	**6**
Grasps side of beam	1
Hugs the beam and one limb falls down from the beam	2
Hugs the beam and two limbs fall down from the beam, or spin on beam (>60 s)	3
Attempt to balance on the beam but falls off (>40 s)	4
Attempt to balance on the beam but falls off (>20 s)	5
Falls off with no attempt to balance or hang on to the beam	6
**Reflexes (blunt or sharp stimulation) absent of:**	**4**
Pinna reflex (head shake after touching the auditory meatus)	1
Corneal reflex (eye blinking after touching the cornea with a cotton)	1
Startle reflex (motor response to a brief noise)	1
Seizures, myoclonus, myodystonia	1
**Maximum points**	**18**

Score: 13–18 severe; 7–12 moderate; 1–6 mild injury.

## Data Availability

Publicly available datasets were analyzed in this study. The bulk transcriptomic datasets are available in the NCBI Gene Expression Omnibus database under accession numbers GSE16561, GSE22255, and GSE58294. The single-cell RNA sequencing dataset is available under accession number GSE174574. The experimental data generated in this study are available from the corresponding author upon reasonable request.

## Supplementary Materials

The following supporting information can be downloaded at: https://www.mdpi.com/article/10.3390/ijms27209035/s1.

## Abbreviations

MCAO	middle cerebral artery occlusion
CSP	Cordyceps polysaccharides
OFT	open-field test
OGD	oxygen-glucose deprivation
tPA	tissue plasminogen activator
TCMPs	traditional Chinese medicinal plants
IL-17A	interleukin-17A
IL-17RA	interleukin-17 receptor A
ACT1	NF-κB activator 1
TRAF6	TNF receptor-associated factor 6
NF-κB p65	nuclear factor kappa-light-chain-enhancer of activated B cells p65 subunit
IL-1β	interleukin-1 beta
IL-6	interleukin-6
IS	ischemic stroke
