# Supplementary material for: Cordyceps Polysaccharides Attenuate Experimental Ischemic Stroke Injury: Association with IL-17/NF-κB Signaling
Source: Int J Mol Sci. 2026 Oct 12;27(20):9035. doi: 10.3390/ijms27209035 (PMC13705458; doi:10.3390/ijms27209035)
Supplement: Supplementary file 1 [file ijms-27-09035-s001.zip › ijms-4528935-supplementary.pdf]

1. Single-cell RNA-seq analysis identified microglia as a brain cell population showing a prominent MCAO-associated increase in an IL-17/NF-κB-related inflammatory module.

To further characterize the brain cell populations associated with MCAO-related IL-17/NF-κB-related inflammatory changes, we analyzed the public mouse MCAO single-cell RNA-seq dataset GSE174574. Quality-control analysis showed comparable distributions of detected genes, UMI counts, and mitochondrial transcript percentages among the included Sham and MCAO samples, supporting the suitability of the dataset for downstream analysis (Supplementary Figure S1A). After quality control, normalization, Harmony-based sample integration, clustering, and marker-based annotation, 13 major brain cell populations were identified, including microglia, CNS-associated macrophages, monocyte-derived cells, neutrophils, lymphocytes, astrocytes, oligodendrocytes, neural progenitor cells, endothelial cells, pericytes/smooth muscle cells, fibroblasts, ependymal/choroid plexus cells, and red blood cells (Supplementary Figure S1B–D). Cells from the Sham and MCAO groups were distributed within the same UMAP space, allowing cell-type-associated changes to be compared between groups (Supplementary Figure S1C). The expression patterns of canonical marker genes further supported the cell-type annotations (Supplementary Figure S1D).

We next calculated an IL-17/NF-κB-related inflammatory module score across all annotated cells and summarized the scores at the biological-sample level. For descriptive between-group comparisons, only cell populations represented by at least 10 cells in each of the six samples were included. Several cell populations showed higher mean module scores in the MCAO group than in the Sham group (Supplementary Figure S1E,F). Neutrophils had the highest absolute module scores in both groups, whereas microglia showed the largest MCAO-associated increase in the group mean score (Δ[MCAO − Sham] = 0.311; Supplementary Figure S1G). Microglia are resident immune cells of the central nervous system and play an important role in the regulation of post-ischemic neuroinflammation. Therefore, considering both the prominent MCAO-associated increase in the microglial module score and the focus of this study on microglia-related inflammatory responses, BV-2 cells were selected as a practical mouse microglial model for subsequent OGD and CSP-intervention experiments. Although neutrophils showed high absolute module scores, their primary cells are short-lived and technically more difficult to maintain reproducibly ex vivo, making them less suitable for the planned cell-based mechanistic experiments.

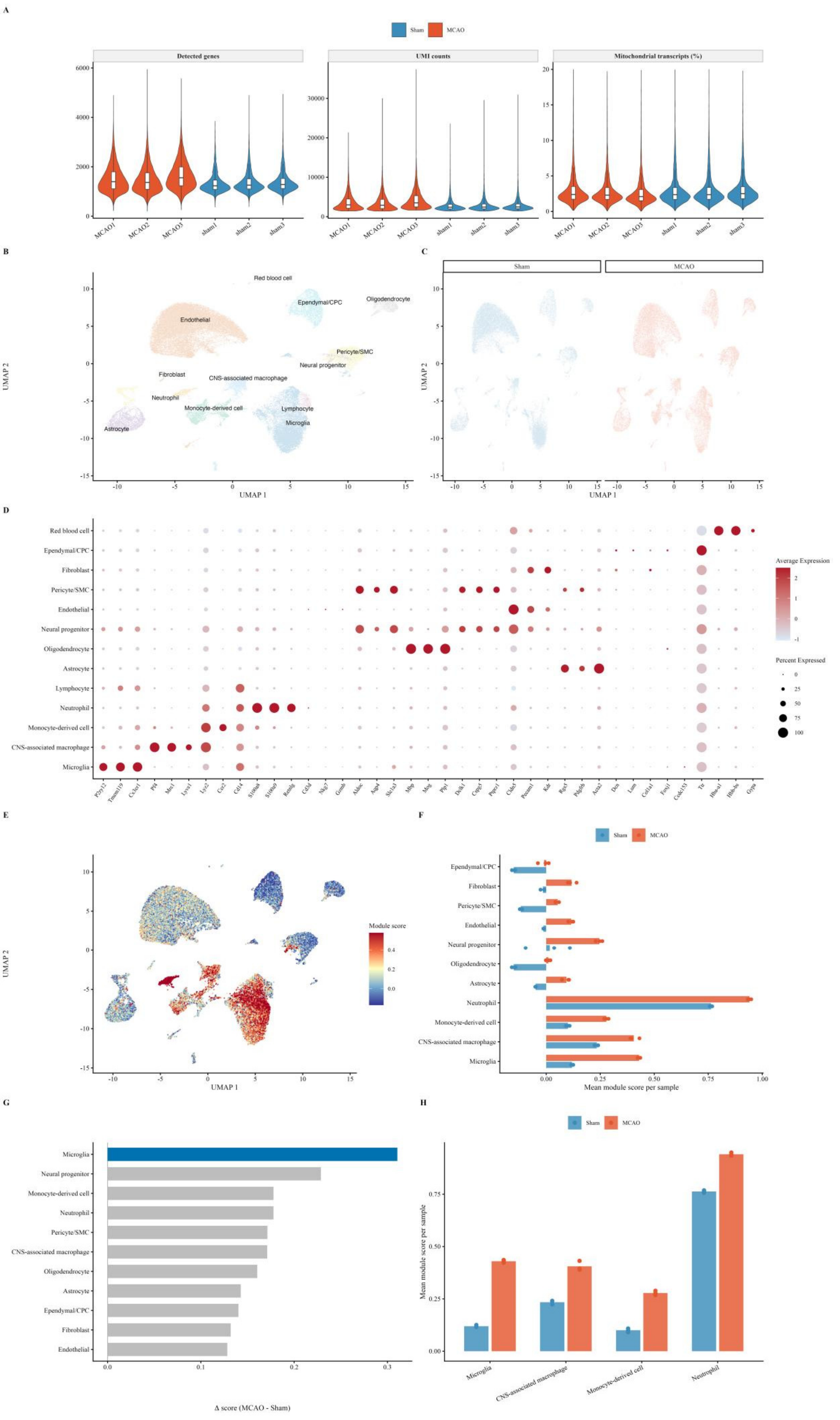
A
Sham
MCAO
Detected genes
UMI counts
Mitochondrial transcripts (%)
MCAO1
MCAO2
MCAO3
sham1
sham2
sham3
B
C
Red blood cell
Ependymal/CPC
Oligodendrocyte
Endothelial
Pericyte/SMC
Neural progenitor
Fibroblast
CNS-associated macrophage
Neutrophil
Monocyte-derived cell
Lymphocyte
Microglia
Astrocyte
UMAP 1
UMAP 2
D
Average Expression
Percent Expressed
P2ry12
Tmem119
Cx3cr1
Pf4
Mrc1
Lyve1
Lyz2
Ccr2
Cd14
S100a8
S100a9
Retnlg
Cd3d
Nkg7
Gzmb
Aldoc
Aqp4
Slc1a3
Mbp
Mog
Plp1
Dcx
Cspg4
Pdgfrb
Cldn5
Pecam1
Kdr
Rgs5
Pdgfrb
Acta2
Dcn
Lum
Col1a1
Foxj1
Cdc20
Ttr
Hba-a1
Hbb-bs
Gypa
E
Module score
F
Mean module score per sample
G
Δ score (MCAO - Sham)
H
Mean module score per sample

Supplementary Figure S1. Single-cell RNA-seq analysis of cell-type-associated IL-17/NF-κB-related inflammatory module scores after MCAO.

(A) Quality-control metrics of the six included single-cell RNA-seq samples, including the number of detected genes, UMI counts, and the percentage of mitochondrial transcripts. (B) UMAP visualization of the annotated brain cell populations, including microglia, CNS-associated macrophages, monocyte-derived cells, neutrophils, lymphocytes, astrocytes, oligodendrocytes, neural progenitor cells, endothelial cells, pericytes/smooth muscle cells, fibroblasts, ependymal/choroid plexus cells, and red blood cells. (C) UMAP visualization of cells from the Sham and MCAO groups. (D) Dot plot showing the expression of canonical marker genes supporting cell-type annotation; dot size indicates the percentage of expressing cells, and color indicates scaled average expression. (E) UMAP visualization of the IL-17/NF-κB-related inflammatory module score across all cells. (F) Sample-level comparison of module scores among sufficiently represented cell populations in the Sham and MCAO groups. Cell populations represented by at least 10 cells in each of the six samples were included. Bars indicate group means, and points represent individual biological samples. (G) Difference in the group mean module score for each sufficiently represented cell population, calculated as MCAO minus Sham. (H) Comparison of module scores in selected myeloid populations, including microglia, CNS-associated macrophages, monocyte-derived cells, and neutrophils. UMI, unique molecular identifier; UMAP, uniform manifold approximation and projection.
